# Electrospun Polyvinyl Alcohol/Chitosan Nanofibers Film Loaded With Artemisia Essential Oil: A Strategy for Extending Shelf Life and Enhancing Quality of Minced Beef

**DOI:** 10.1002/fsn3.71330

**Published:** 2025-12-12

**Authors:** Amirhossein Nasiri, Tayebeh Zeinali, Elham Ansarifar

**Affiliations:** ^1^ Student Research Committee Birjand University of Medical Sciences Birjand Iran; ^2^ Department of Food Hygiene and Nutrition, School of Health, Geriatric Health Research Center Birjand University of Medical Sciences Birjand Iran; ^3^ Department of Food Hygiene and Nutrition, School of Health, Social Determinants of Health Research Center Birjand University of Medical Sciences Birjand Iran

**Keywords:** active packaging, artemisia essential oil, electrospinning, minced beef meat

## Abstract

Meat, due to its high‐water activity and nutrient‐rich composition, is highly prone to microbial spoilage, especially after mincing, which accelerates contamination and reduces shelf life. This study aimed to develop an active packaging film by incorporating Artemisia essential oil (AEO) into electrospun chitosan/polyvinyl alcohol (CH + PVA) nanofibers films to extend the shelf life and preserve the quality of minced beef. Electrospinning was employed to fabricate CH + PVA nanofiber films, with and without AEO, and their morphology was characterized using scanning electron microscopy (SEM). Minced beef samples were packaged in control, CH + PVA, and CH + PVA + AEO films and stored at 4°C for 18 days. The CH + PVA + AEO film significantly reduced (*p* < 0.05) total viable count (56.91%), psychrotrophic count (34.53%), pH (53.85%), peroxide value (PV) (40.91%), total volatile nitrogen bases (TVB‐N) (28.58%), thiobarbituric acid (TBA) (31.46%), and free fatty acids (FFA) (54.66%) compared with the control. Moreover, sensory evaluation confirmed that CH + PVA + AEO film preserved color, texture, odor, and overall acceptability, extending acceptability to 12 days (*p* < 0.05). In conclusion, the incorporation of AEO into electrospun CH + PVA nanofiber film provides a sustainable and effective strategy for developing active packaging film, offering a natural alternative to synthetic preservatives for improving the shelf life and quality of minced beef.

## Graphical abstract

This study demonstrated that chitosan/polyvinyl alcohol nanofiber films containing Artemisia essential oil (CH + PVA + AEO) effectively reduced microbial and chemical spoilage in minced beef. Significant decreases (*p* < 0.05) were observed in microbial counts, pH, peroxide value, TVB‐N, TBA, and FFA compared with the control. Sensory evaluation confirmed better preservation of color, texture, and odor, extending consumer acceptability to 12 days. Overall, incorporating AEO into electrospun CH + PVA films offers a natural and sustainable alternative to synthetic preservatives for improving meat shelf life.

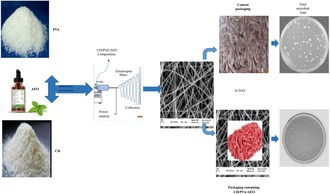

## 1 Introduction

Meat is one of the richest sources of protein, but it also provides a suitable environment for the growth of various microorganisms. This is due to its physical and chemical characteristics, including high water activity, moderate pH, and a diverse and appropriate combination of nutrients (e.g., protein, vitamins, minerals, etc.; Ercolini et al. 2011; Sayadi et al. 2022). Meat processing, such as mincing, increases the surface area, which in turn increases oxygen penetration, mixes the surface contamination with the deep part, and ultimately accelerates spoilage (Surendhiran et al. 2020). Cold storage is a common method for increasing the shelf life of minced meat. To further enhance shelf life, various methods can be employed, including the use of active packaging, modified atmosphere packaging, plant extracts or essential oils, organic acids, and edible films and coatings. These methods significantly contribute to preserving the quality and safety of minced meat products (Chawla et al. 2021; Lotfy et al. 2023).

Plant essential oils have become increasingly important as natural preservatives in the food industry due to their antimicrobial properties. They not only inhibit microbial growth but also enhance the sensory qualities of food products, making them a preferred alternative to synthetic preservatives (Yaghoubi et al. 2021). *Artemisia essential oil*, scientifically known as *Artemisia sieberi Besser*, is increasingly recognized in the food industry as a natural preservative. Its antimicrobial and antioxidant effects are attributed to key active compounds such as camphor, 1,8‐cineole, and α‐pinene (Meng et al. 2023). Studies have demonstrated its antimicrobial activity against various microorganisms, including gram‐negative and gram‐positive bacteria (Chebbac et al. 2023). Overall, *Artemisia sieberi* essential oil is a promising alternative to synthetic preservatives, potentially enhancing both the safety and sensory qualities of food products (Alirezalu et al. 2022).

The production of polymer nanofibers, particularly through electrospinning, is a key area of nanotechnology with significant applications in food packaging (Rezaei Soulegani et al. 2021). Electrospinning enables the creation of nanofibers ranging in diameter from micrometers to nanometers, characterized by high porosity and specific surface area. This technique is effective for creating active packaging materials by encapsulating bioactive compounds, such as plant essential oils. This enhances functionality through controlled release and protection against chemical degradation (Asghari Ghajari et al. 2021). Key benefits of electrospinning include efficient microcoating, extended storage time, cost‐effectiveness, and adaptability to high temperatures and environmental pressures (Liu et al. 2021).

Chitosan, a linear polysaccharide and deacetylated chitin derivative, is gaining significant attention as an antimicrobial preservative in food packaging due to its non‐toxic, biocompatible, and biodegradable nature (Abdelrazek et al. 2010; Sharafati Chaleshtori et al. 2016). Its inherent antibacterial and antifungal properties, along with its ability to form films, make chitosan an ideal candidate for antimicrobial packaging applications (Surendhiran et al. 2020). However, the use of chitosan in the food packaging industry faces challenges such as weak mechanical properties in moist environments and permeability to gases and water vapor (Moalla et al. 2021). Research indicated that combining chitosan with synthetic polymers like poly vinyl alcohol (PVA) can enhance its mechanical properties and overall performance (Yaghoubi et al. 2021). PVA is a biodegradable, non‐toxic thermoplastic with excellent film‐forming capabilities (Moradinezhad et al. 2023; Tatlisu et al. 2019). The resulting chitosan‐PVA composite films exhibit improved mechanical and physical properties compared to either polymer alone, offering better stability and controlled release of encapsulated compounds (Tripathi et al. 2009). This combination thus represents a promising approach to develop effective antimicrobial packaging solutions that increase shelf life and food safety.

Therefore, this study aims to investigate the effectiveness of packaging containing CH + PVA electrospun nanofibers, incorporating encapsulated *Artemisia essential oil* (AEO), in maintaining the sensory characteristics and increasing the shelf life of minced beef.

## 2 Materials and Method

### 2.1 Materials

Polyvinyl alcohol (PVA, Mw ≈130,000 Da) and chitosan (CH, degree of deacetylation 75%–85%, Mw = 190,000–310,000 Da) were purchased from Sigma‐Aldrich (USA). Glacial acetic acid and other analytical grade solvents were obtained from Merck (Germany). “Artemisia essential oil (AEO) was obtained pre‐prepared from Tabib Darou Company, where it had been extracted from the aerial parts of Artemisia sieberi collected in Khorasan Province, Iran, using hydrodistillation with a Clevenger‐type apparatus for 3 hours. Upon receipt, the oil was filtered, and stored in sealed dark glass vials at 4°C until further use.”

The chemical composition of AEO was determined by GC–MS analysis. The predominant constituents were camphor (21.48%), α‐thujone (20.73%), β‐thujone (9.64%), α‐ionol (13.5%), artemisia alcohol (3.05%), cis‐verbenol (3.18%), p‐menth‐1,5‐dien‐8‐ol (3.98%), and 1,8‐cineole (3.42%), with minor components such as α‐pinene, camphene, cymene, santolina alcohol, and bornyl acetate present in lower concentrations (< 3%). These bioactive compounds are responsible for the antimicrobial and antioxidant activities of the essential oil.

### 2.2 Solution Preparation

A solution of CH + PVA was created by dissolving 2.5% Chitosan polymer and 10% PVA polymer in distilled water with 1% glacial acetic acid, which was stirred for 2 h at 80°C to achieve a homogeneous mixture. *Artemisia essential oil* (2% w/v) was incorporated into the CH + PVA solution and allowed to mix for 24 h.

### 2.3 Electrospinning

An electrospinning machine with a high‐voltage power supply (ES‐1000 model, Nanoscale Technologies Co., Iran) was used to generate nanofibers. The device's voltage was connected to a stainless‐steel capillary needle, while a polymer solution was placed in a 5 mL syringe and delivered to the needle via a narrow tube using a digitally controlled pump. The collector in the electrospinning setup consisted of a metal plate coated with aluminum foil. Device parameters, including the applied voltage, feeding rate, and the distance between the needle and the collector plate, were set at 15 kV, 0.5 mL/h, and 15 cm, respectively (Moradinezhad et al. 2023).

### 2.4 Scanning Electron Microscope (SEM)

The morphology of the electrospun fibers was analyzed using a scanning electron microscope (Leo 1450VPSEM) operating at a voltage of 20 kV. Using ImageJ V1.48 software, the diameter of 40 fibers was randomly measured. Finally, the average of these data was considered as the average fiber diameter of that image (Moalla et al. 2021).

### 2.5 Minced Meat Preparation and Active Packaging

Minced beef was acquired from a local store in Birjand, Khorasan Province, and stored in insulated polystyrene boxes on ice for transport to the laboratory, which occurred within 1 h of purchase. Subsequently, to evaluate the impact of CH + PVA nanofiber films, both with and without AEO, on the shelf life of minced beef during refrigeration, the study was structured using a factorial design. Minced meat was stored in polyethylene containers (measuring 11.5 × 9.5 × 6.2 cm) that were thoroughly sterilized, divided into three groups with lids securely fitted (about 150 g per package). A total of 3 types of packaging containing nanofibers with and without essential oil and a control packaging (without nanofibers and essential oil) were used.

1. Control group: Minced meat was placed in containers with lids.
2. CH + PVA group: Minced meat was placed in active packaging with film (CH + PVA nanofiber without AEO) installed on the lid (its inner part) (No contact with nanofibers).
3. CH + PVA + AEO group: Minced meat was placed in active packaging with film (CH + PVA nanofibers containing AEO) installed on the lid (its inner part) (No contact with nanofibers).

Minced meat packages were categorized into three groups and stored under refrigerated conditions (4°C ± 0.5°C and 85% relative humidity). The quality parameters of the minced meat were evaluated on days 0, 3, 6, 9, 12, 15, and 18 of storage, with assessments conducted independently in triplicate.

### 2.6 Attributes Evaluated in Minced Meat

#### 2.6.1 Microbial Analysis

During each sampling instance, 10 g of minced beef was combined with 90 mL of sterile 0.1% peptone water and homogenized using a laboratory mixer (stomacher 400, Interscience, France) for a duration of 2 min. Subsequently, decimal dilutions were prepared in a 0.1% peptone water solution, and these dilutions were either spread or poured onto the suitable agar (plate count agar) medium. Microbial assessments encompassed the determination of total viable count (TVC) and psychrotrophic count (PTC), which were evaluated on plate count agar at 37°C for 24 to 48 h and at 7°C for a period of 10 days, respectively. Finally, TVC and PTC counts were expressed as Log cfu/g (Bagheri and Aryaee 2022).

#### 2.6.2 pH

To measure pH (After calibrating the pH meter), 5 g of minced meat from each treatment was homogenized in 45 mL of distilled water for 1 min. The pH value of the samples was then read at room temperature using a digital pH meter (Sartorius, Germany) (Fattahian et al. 2020).

#### 2.6.3 Peroxide Value (PV)

To measure the PV, about 5g of fat extracted from minced meat samples, were mixed with 30 mL of chloroform/acetic acid solution (3:2 ratio), and 0.5 mL of saturated potassium iodide was added. The resulting mixture was placed in the dark for 1 min. In the next step, 0.5 mL of 1% starch solution was added as a reagent, and the resulting mixture was vigorously shaken. Finally, it was titrated with 0.01 normal sodium thiosulfate until the blue color disappeared (Ahmadi and Alishahi 2018).

#### 2.6.4 Total Volatile Nitrogen Bases (TVB‐N)

To determine the total volatile nitrogen bases (TVB‐N), 10 g of minced beef was mixed with 2 g of magnesium oxide (MgO) and 300 mL of distilled water in a Kjeldahl flask. The flask was connected to the Kjeldahl distillation apparatus. A 250 mL Erlenmeyer flask containing 25 mL of 2% boric acid solution (with a few drops of methyl red indicator) was attached to the end of the apparatus. The mixture was distilled for 45 min, during which the boric acid solution changed color from red to yellow, indicating the absorption of volatile nitrogen bases. After distillation, the boric acid solution was titrated with 0.1 N sulfuric acid (H_2_SO_4_) until the solution returned to a red color. The TVB‐N content was then calculated in milligrams per 100 g of minced meat (Ahmadi and Alishahi 2018).

#### 2.6.5 Thiobarbituric Acid (TBA)

To measure thiobarbituric acid (TBA), 200 mg of fat, extracted from 2 g minced beef sample using hexane, was placed into a 25 mL volumetric flask and diluted to volume with 1‐butanol solution. Next, 5 mL of this solution was transferred to a dry, capped tube, and 5 mL of TBA reagent was added to it. The tubes were placed in a hot water bath at 95°C for 2 h. After heating, the tubes were cooled to room temperature. Finally, the absorption of the samples was measured using a spectrophotometer at a wavelength of 530 nm. The TBA value was calculated in terms of milligrams of malondialdehyde (MDA) equivalent per kilogram of meat sample (mg MDA/kg meat) (Pilmal et al. 2018).

#### 2.6.6 Free Fatty Acid (FFA)

To determine the amount of free fatty acids (FFA), approximately 3 g of the extracted oil from sample was weighted and transferred into an Erlenmeyer flask. Then, 30 mL of neutralized ethyl alcohol and 2–3 drops of phenolphthalein indicator were added to the flask. The mixture was titrated with 0.1 N sodium hydroxide (NaOH) solution until persistent pale pink color appeared, indicating the endpoint of the titration. Finally, the acidity was calculated in terms of oleic acid percentage (Bagheri and Aryaee 2022).

#### 2.6.7 Color Parameters

To examine the impact of treatments on the surface color alterations of minced meat samples, the following methodology was adopted: The procedure encompassed image capture, color profiling and calculation, and conversion of images into L*, a*, b* units. Uniform sample lighting conditions were maintained in line with a prior study (Noshad et al. 2015). Adobe Photoshop was utilized to enhance background contrast and facilitate segmentation. Subsequently, the images were transformed into L*, a*, b* units to ensure consistent color perception and approximate the Euclidean distance between colors. Image assessment was conducted using Image J software (National Institutes of Health, Bethesda, MD, USA) version 1.40 g (Noshad et al. 2015).

#### 2.6.8 Sensory Analysis

To conduct the sensory evaluation test, a panel of 20 evaluators was selected based on their availability, interest, and previous experience in sensory evaluation of meat products. Prior to the formal evaluation, the panelists underwent a training process. The training consisted of two 1‐h sessions. In the first session, panelists were introduced to the 9‐point hedonic scale and the specific attributes to be evaluated (odor, color, texture, appearance, and overall acceptability). They were familiarized with reference standards for fresh and spoiled minced beef, focusing on the detection of off‐odors (e.g., sour, putrid) and visual spoilage indicators. The second session was practical training where panelists evaluated representative fresh and intentionally aged beef samples to standardize their scoring criteria and ensure consistency in their assessments. The panel consisted of 10 men and 10 women, with an average age of 40 years. All participants provided informed consent before participating in the test. Seven sessions were conducted for sensory evaluation (Days 0, 3, 6, 9, 12, 15, and 18). They evaluated the odor, color, texture, appearance, and overall acceptability of each sample (from each treatment). Their opinions were recorded using a pre‐prepared questionnaire based on a standard 9‐point hedonic scale (where 1 = dislike extremely, 5 = neither like nor dislike, and 9 = like extremely) (Esmaeili and Khodanazary 2021).

### 2.7 Statistical Analysis

The data was analyzed using a completely randomized design to verify the results obtained from various tests. Initially, the data's normality was assessed through the Kolmogorov–Smirnov test. To ascertain variations among the mean values (with a minimum of three replicates for each experiment), Student t Test was conducted following the analysis of variance at a significance level of *p* < 0.05. In cases where the data did not exhibit normal distribution, nonparametric tests were employed as alternatives. Statistical analysis was carried out using the SAS JMP PRO statistical software program, version 18.0.1.

## 3 Results and Discussion

### 3.1 SEM

**FIGURE 1:**
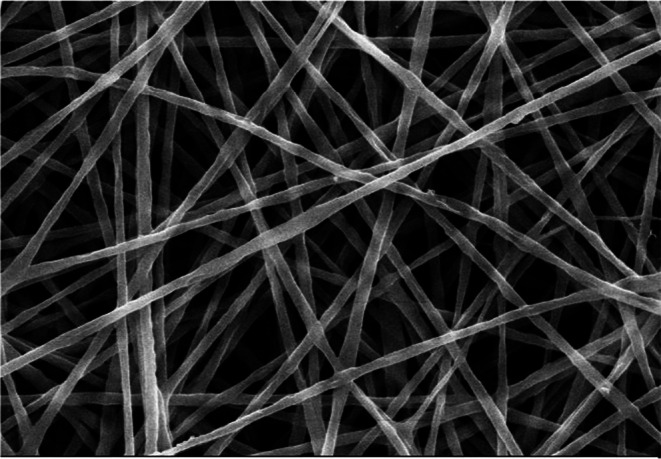
Scanning electron microscope image of the electrospun CH + PVA nanofibers incorporated with *Artemisia* essential oil (AEO).

The morphology of the electrospun nanofibers loaded with Artemisia essential oil (AEO) was examined using scanning electron microscopy (SEM). The resulting micrograph (Figure 1) reveals that the incorporation of AEO into the polymer matrix led to the formation of nanofibers with a smooth, uniform, bead‐free morphology. The fibers were continuous and well‐formed, indicating good compatibility between the essential oil and the polymeric blend as well as successful encapsulation during the electrospinning process. Previous studies on chitosan‐PVA composite fibers have consistently reported favorable morphological characteristics. For instance, Tripathi et al. (2009) observed that chitosan‐PVA fiber films intended for food packaging exhibited a relatively smooth, homogeneous, and crack‐free morphology with good structural integrity. Similarly, Rezaei Soulegani et al. (2021) confirmed that composite fibers composed of chitosan and polyvinyl alcohol in a 70:30 ratio showed uniform morphology, further supporting the structural integrity and consistency of CH‐PVA fiber films.

The average diameter of the nanofibers produced from the CH + PVA solution containing AEO was found to be 450 ± 30 nm. This diameter range can be attributed to changes in the solution's properties, such as viscosity and electrical conductivity, upon the incorporation of the essential oil. Specifically, the addition of AEO reduces the electrical conductivity of the polymer solution, which decreases the charge density of the electrospinning jet. This reduction in charge density results in less electrostatic stretching of the jet during the electrospinning process, ultimately leading to the formation of fibers with a larger diameter. This observation is consistent with findings from similar studies; Moradinezhad et al. (2023) reported that adding *Zataria multiflora* essential oil increased the diameter of nanofibers. Furthermore, Rezaei et al. (2021) attributed an increase in fiber diameter from the addition of *Artemisia sieberi* essential oil to chitosan nanofibers to a decrease in electrical conductivity.

### 3.2 Assessment of Minced Beef Meat Quality

#### 3.2.1 Microbial Analysis

**FIGURE 2:**
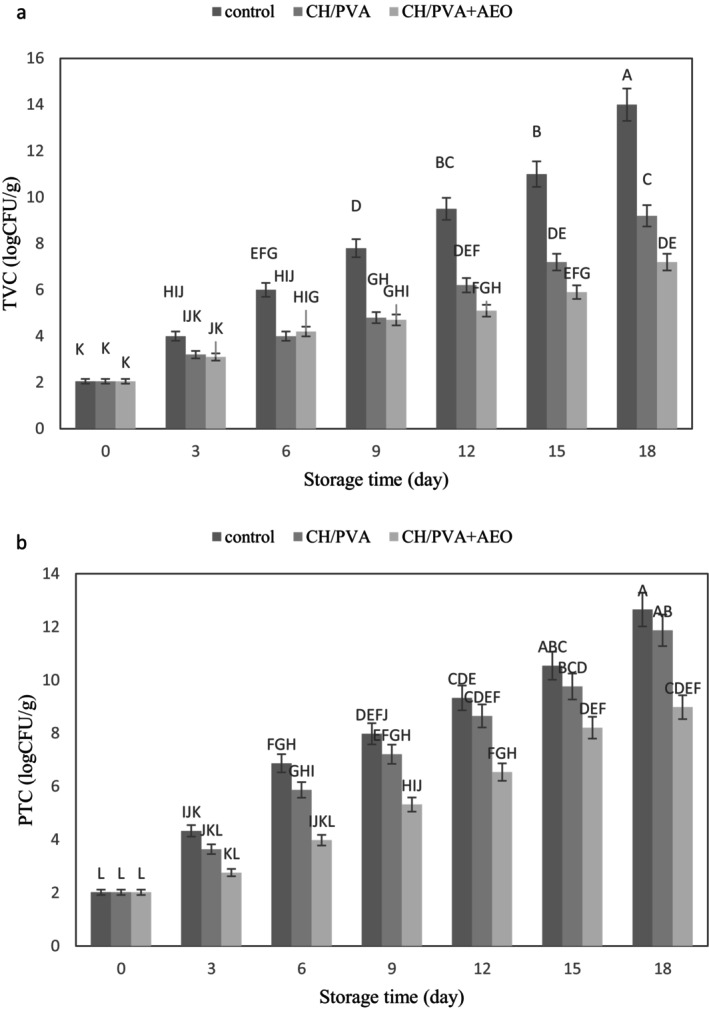
Total viable count (TVC) (a) and psychrotrophic count (PTC) (b) of minced beef meat packaged in packaging control, CH + PVA, CH + PVA + AEO during 18 days of storage at 4°C. Different letters indicate significant differences (*p* < 0.05).

The total viable count (TVC) serves as an indicator of the sanitary quality of a product, determining its suitability for consumption (Bagheri and Aryaee 2022; Osman et al. 2020). The results related to changes in TVC are illustrated in Figure 2a. As expected, the total bacterial counts increased across all treatments during the 18‐day refrigerated storage period. Among the various treatments, the control sample exhibited the highest increase in microbial load, which is consistent with findings from other researchers (Pilmal et al. 2018; Venkatachalam and Lekjing 2020). According to Figure 2a, the initial TVC in fresh minced meat was 2.05 log CFU/g for all treatments. With prolonged storage time, the TVC increased significantly. By the end of the 18th day of storage, the TVC for the control sample reached 14 log CFU/g, while it was 9.2 log CFU/g for the CH + PVA treatment and 7.2 log CFU/g for the CH + PVA + AEO treatment. Based on published data from Ercolini et al. (2011), the acceptable upper limit of TVC for all meats and meat products is 7 log CFU/g. In this study, the TVC of the control sample exceeded this threshold after 9 days, reaching 7.8 log CFU/g, but the CH + PVA + AEO treatment exceeded the threshold after 18 days. Due to environmental conditions and the biological composition of meat, it is challenging to store it even at cold temperatures, leading to chemical and microbial spoilage (Ercolini et al. 2011). The lower total bacterial count observed in the treatment containing AEO compared to other treatments is likely related to the antimicrobial compounds present in this essential oil (Rezaei et al. 2021).

Counting the number of psychrotrophic bacteria (PTC) is an important indicator of meat safety and quality, as these microorganisms are primarily responsible for meat spoilage at cold temperatures (Bagheri and Aryaee 2022). According to Figure 2b, PTC increased in all investigated treatments during the 18‐day refrigerated storage period. Among the different treatments, the control sample exhibited a more pronounced increase in microbial load throughout the storage period, consistent with findings from other researchers (Venkatachalam and Lekjing 2020). The initial PTC value in fresh minced meat for all samples was 2.02 log CFU/g. As expected, with increasing storage time, the PTC content rose significantly (*p* < 0.05). By the end of the 18th day of storage, PTC values were recorded at 12.65 log CFU/g for the control sample, 11.87 log CFU/g for the CH + PVA treatment, and 8.98 log CFU/g for the CH + PVA + AEO treatment. The results indicated that the PTC levels in treatment CH + PVA + AEO were significantly less than the control sample throughout the storage period (*p* < 0.05). This reduction may be attributed to the antimicrobial properties of CH and AEO, which aligns with studies conducted by other researchers (Fattahian et al. 2020). In support of this study, Rezaei et al. (2021) demonstrated that incorporating *Artemisia Sieberi* essential oil into CH nanofibers enhances their antimicrobial properties against both gram‐negative and gram‐positive bacteria. Specifically, *Artemisia Dashti* essential oil contains phenolic compounds, saponins, tannins, alpha‐pinene, and camphor, which can disrupt bacterial cell walls. Bagheri and Aryaee (2022) noted that using CH in combination with nettle essential oil and basil gum effectively reduces microbial parameters in hamburger products. The reduction in PTC values observed in treatments containing CH may be due to interactions between positively charged amine groups in CH and negatively charged macromolecules on microbial cell surfaces (Nowzari et al. 2013). In a similar study, Venkatachalam and Lekjing (2020), reported that chitosan‐based edible film with *clove* essential oil and nisin reduced TVC and PTC levels in fish meat samples during refrigeration. The mechanism of action of CH seems to be related to its interaction with the negatively charged microbial cell membrane and its function as a barrier against bacterial growth.

#### 3.2.2 pH

**FIGURE 3:**
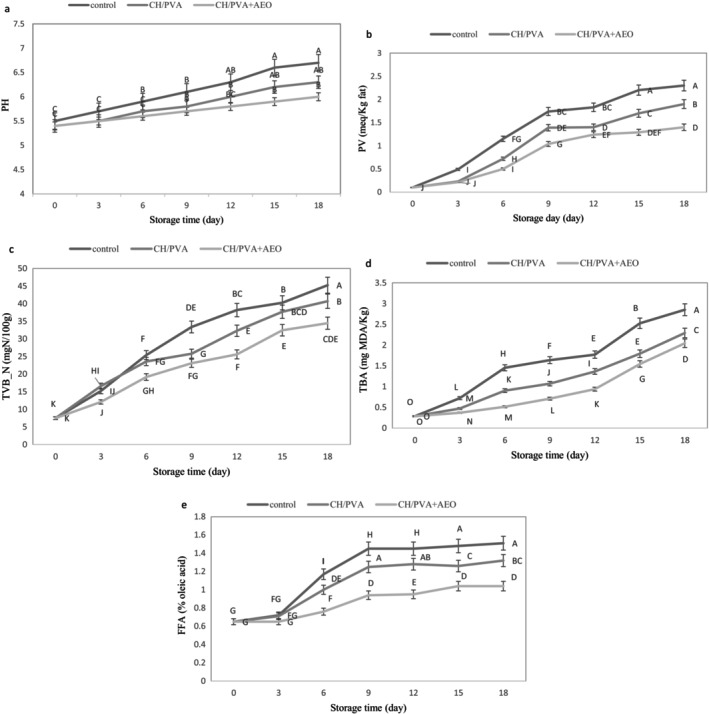
Changes in pH (a), peroxide value (PV) (b), total volatile nitrogen bases (TVB‐N) (c), thiobarbituric acid (TBA) (d), free fatty acid (FFA) (e) of minced beef packaged in packaging control, CH + PVA, CH + PVA + AEO during 18 days of storage at 4°C. Different letters indicate significant differences (*p* < 0.05).

pH is a critical parameter for indicating the decline in meat quality during storage. An increase in pH negatively impacts product quality throughout the storage period (Fattahian et al. 2020; Venkatachalam and Lekjing 2020). Figure 3a presents the changes in pH of minced meat over an 18‐day storage period at refrigeration (4°C). As anticipated, pH levels increased in all treatments over time. The rise in pH during refrigerated storage of minced meat can be attributed to microbial activity and biochemical processes (Konuk Takma and Korel 2019). Osman et al. (2020) and Venkatachalam and Lekjing (2020) reported that bacteria produce metabolites such as ammonia and amines as they proliferate, leading to an increase in meat pH. Furthermore, research by Ahmadi and Alishahi (2018) showed that the activity of proteolytic and autolytic enzymes of spoilage bacteria can increase pH. Among all samples, the control sample showed the highest increase in pH during the 18‐day refrigeration period. The initial pH of all samples was on average 5.4, with final pH values recorded at 6.7 for the control sample, 6.3 for the CH + PVA treatment, and 6.0 for the AEO + CH + PVA treatment. The lower pH value observed in the AEO + CH + PVA treatment can be attributed to the antimicrobial properties of the *Artemisia essential oil* (Rezaei Soulegani et al. 2021). In related research, Yaghoubi et al. (2021) reported that the reason for the lower pH observed in coated chicken meat samples compared to control samples could be due to the antibacterial properties of chitosan and *Artemisia* essential oil.

#### 3.2.3 Peroxide Value

The peroxide value (PV) of minced beef serves as an indicator of lipid oxidation, which is a primary contributor to rancidity in meat products (Venkatachalam and Lekjing 2020). Lipid oxidation leads to the formation of peroxides and hydroperoxides, which are the primary products of the oxidation reaction (Ahmadi and Alishahi 2018). The PV quantifies the concentration of these peroxides and is used to assess the freshness and quality of meat (Pilmal et al. 2018). As illustrated in Figure 3b, the PV increased in all samples during the 18‐day storage period. The initial PV of the samples was recorded, on average, at 0.1 meq/kg fat. However, the PV increased steadily, reaching a maximum of 2.3 meq/kg fat for the control sample by day 18. This pattern is consistent with findings from Sayadi et al. (2022), where a slowdown in the PV increase from the 12th day onward, initiated by the breakdown of peroxide compounds, was observed (Figure 3b). The PV value of the control sample was significantly higher than other samples during the storage period (*p* < 0.05). By the 18th day, the PVs for the control, CH + PVA, and CH + PVA + AEO treatments were recorded at 2.3, 1.9, and 1.4 meq/kg fat, respectively. These results indicate that nanofiber‐containing packaging, particularly the antimicrobial combinations of CH + PVA and CH + PVA + AEO, significantly (*p* < 0.05) reduced the peroxide value of minced beef during the 18‐day storage period at 4°C. This reduction may be attributed to the strong antioxidant and antibacterial properties of AEO, which help reduce microbial load and spoilage (Lotfy et al. 2023). Incorporating essential oils, such as those derived from *Artemisia*, into nanofibers can significantly enhance their antioxidant properties significantly (*p* < 0.05). Essential oils have demonstrated considerable antioxidant activity, which aids in reducing the formation of peroxides in meat. This dual approach of utilizing both chitosan and essential oils in nanofiber packaging creates a synergistic effect, leading to improved preservation of minced beef. The addition of essential oils, such as *Citrus limon* and *cinnamon*, further enhances the antimicrobial efficacy of CH‐based films, resulting in lower levels of TVB‐N and TBA, which are indicators of spoilage and oxidation (Pilmal et al. 2018). Studies indicate that CH nanoemulsions combined with essential oils can effectively maintain the quality of refrigerated minced meat, significantly lowering peroxide values and enhancing sensory characteristics (Farajzadeh et al. 2016). In addition, active packaging films made of CH nanofibers and cellulose have shown significant reductions in microbial growth and oxidation, thereby increasing the shelf life of beef during refrigerated storage (Bagheri and Aryaee 2022).

#### 3.2.4 Total Volatile Base Nitrogen

The total volatile nitrogen (TVB‐N) is a crucial indicator for assessing the freshness of meat and its products, particularly minced meat, as it reflects the rate of spoilage, decomposition, and protein breakdown (Bagheri and Aryaee 2022; Pezeshk et al. 2011). As shown in Figure 3c, as predicted, TVB‐N levels increased in all treatments during the 18‐day storage period at 4°C. The rise in TVB‐N during storage can be attributed to the activities of spoilage bacteria, as well as the action of various internal enzymes involved in processes such as amine oxidation, removal of free amino acids, and degradation of nucleotides (Sayadi et al. 2022).

Other studies support these findings; Bagheri and Aryaee (2022) demonstrated that in fresh hamburger, the level of TVB‐N increased from 11.2 to 47.65 mg/100 g over a 12‐day period at 4°C, indicating spoilage. Similarly, Pezeshk et al. (2011) found that fish meat stored in refrigeration, TVB‐N levels rose from 12 mg/100 g to 39 mg/100 g by the 20th day of storage, further confirming the progression of spoilage. In this study, the initial TVB‐N content of minced meat across all treatments was measured, on average, at 7.5 mg N/100 g (Figure 3c). As expected, TVB‐N values increased with prolonged storage time. The optimal limit for total volatile nitrogenous bases in meat and its products is set at 25 mg N per 100 g of meat (Fattahian et al. 2020). Notably, in the control sample, the TVB‐N level exceeded the threshold for spoilage initiation (33.39 mg N/100 g) after just 9 days of storage.

In the treatments CH + PVA + AEO and CH + PVA, the threshold for TVB‐N was reached after the 12‐day storage. The lower levels of volatile nitrogen in the treatment CH + PVA + AEO compared to the control sample and the CH + PVA sample can be attributed to the antibacterial effects of *Artemisia essential* oil, which disrupts bacterial cell membranes and subsequently limits TVB‐N production (Meng et al. 2023). Additionally, electrospun CH + PVA nanofibers create a physical barrier that helps absorb excess moisture and prevent oxidation. The synergistic effect of combining CH + PVA nanofibers with AEO may enhance the overall preservation efficacy on minced meat. CH + PVA nanofibers have the capacity to encapsulate essential oils, allowing for controlled release that maintains antimicrobial activity over time, which can potentially reduce TVB‐N levels by limiting microbial activity (Bagheri and Aryaee 2022). In confirmation of this study, the research results of Pilmal et al. (2018) demonstrated that fish meat coated with chitosan CH and CH combined with cinnamon essential oil resulted in a significant decrease in TVB‐N levels in fish meat samples stored under refrigeration (*p* < 0.05). Additionally, the findings from Yaghoubi et al. (2021) indicated that CH coatings combined with *Artemisia essential oil* delayed the development of TVB‐N reactions by inhibiting bacterial growth in fresh chicken during refrigerated storage.

#### 3.2.5 Thiobarbituric Acid

Thiobarbituric acid (TBA) is utilized to measure the levels of secondary oxidation products, particularly aldehydes, based on malondialdehyde (MDA) content, and serves as an indicator of fat oxidation (Venkatachalam and Lekjing 2020). Malondialdehydes are generated through the oxidation of hydroperoxides into aldehydes and ketones (Bagheri and Aryaee 2022). As illustrated in Figure 3d, TBA levels increased across all treatments during the storage of minced meat at 4°C. This upward trend in TBA during the storage period may be attributed to the oxidation of unsaturated fatty acids, the production of volatile compounds, and an increase in free iron within the muscle (Ghebleh and Sever 2020). Supporting this study, Bagheri and Aryaee (2022) showed that the combination of chitosan nanofibers with nettle essential oil and basil seed gum effectively slows down the increasing trend of TBA index compared to the control sample in fresh hamburger during storage. This effect is likely due to the antioxidant and antimicrobial properties of CH, basil gum, and nettle essential oil, which inhibit free radicals and chelate iron ions.

The initial TBA level for all minced beef treatments, on average, was 0.285 mg MDA/kg. As anticipated, TBA levels increased throughout the 18‐day storage period for all treatments, with a more pronounced increase observed in the control samples. By the end of the storage period, the control sample exhibited the highest TBA level at 2.852 mg MDA/kg, while the treatment with CH + PVA + AEO recorded the lowest TBA level at 2.044 mg MDA/kg. Fattahian et al. (2020), found that utilizing CH coatings in conjunction with cumin essential oil effectively reduces fat oxidation and TBA levels, ultimately extending the shelf life of beef. The mechanism this reduction is attributed to CH coating's ability to prevent oxygen infiltration, thereby slowing down the rate of oxygen penetration from the surrounding space to the surface of the meat and consequently reducing fat oxidation (Bagheri and Aryaee 2022; Venkatachalam and Lekjing 2020). In a similar study, Ghebleh and Sever (2020) demonstrated that CH coatings exhibit antioxidant activity, which is enhanced when essential oils are incorporated, ultimately leading to a reduction in the increasing trend of TBA in chicken. Karimkhani and Bagheri Sales (2021) reported that *Artemisia* extract possesses antimicrobial and antioxidant properties due to its high content of phenolic and flavonoid compounds. These compounds contribute to a reduction in TBA levels in hamburgers by inhibiting fat oxidation and neutralizing free radicals.

#### 3.2.6 FFA

Free fatty acids (FFA) are produced during storage as a result of the enzymatic hydrolysis of lipid esters (Khare et al. 2016). While the presence of free fatty acids does not directly cause quality loss, an increase in their concentration can lead to protein denaturation, heightened fat oxidation, the development of off‐flavors, and a subsequent decline in product quality (Bagheri and Aryaee 2022). Changes in FFA levels in minced meat over an 18‐day storage period at 4°C are illustrated in Figure 3e, FFA levels increased across all treatments during the storage period, aligning with findings from other researchers (Esmaeili and Khodanazary 2021; Venkatachalam and Lekjing 2020). The increase in FFA is attributed to the hydrolytic oxidation of fats mediated by internal enzymes such as lipase and phospholipase (Nowzari et al. 2013). The initial FFA content across all samples, on average, was 0.65% oleic acid. According to the results, the control exhibited the highest FFA levels during the 18‐day storage period. By the end of the 18th day, the final FFA levels were recorded as 1.51% for the control, 1.32% for the CH + PVA treatment, and 1.04% for the CH + PVA + AEO treatment. The significant (*p* < 0.05) difference in FFA levels between the control sample and other treatments highlights the effectiveness of antioxidants present in essential oils and polymers in mitigating hydrolytic spoilage (Esmaeili and Khodanazary 2021). The results of this research align with those of Bagheri and Aryaee (2022), who showed that the low levels of FFA in the treatments containing chitosan (CH) and nettle essential oil in hamburger can be attributed to the presence of phenolic compounds in the essential oil. These phenolic compounds not only inhibit free radicals but also prevent the penetration of oxygen into the packaging, thereby mitigating oxidative spoilage. In a similar study, Esmaeili and Khodanazary (2021) demonstrated that the lowest levels of FFA were observed in groups treated with CH containing *Artemisia dracunculus* essential oil compared to untreated groups. They attributed this reduction to the high antioxidant and antimicrobial activity of AEO, which effectively inhibits the enzymatic hydrolysis of esterified lipids. Furthermore, Khare et al. (2016) reported that CH coatings containing cinnamon essential oil significantly reduced FFA production in chicken nuggets during refrigerated storage. This reduction in FFA can be ascribed to the chelating activity of CH, which forms bonds with metal ions and ultimately prevents microbial growth. Additionally, CH has been identified as an inhibitor of enzyme activity, thereby reducing the oxidation processes of fats (Venkatachalam and Lekjing 2020).

#### 3.2.7 Color

**FIGURE 4:**
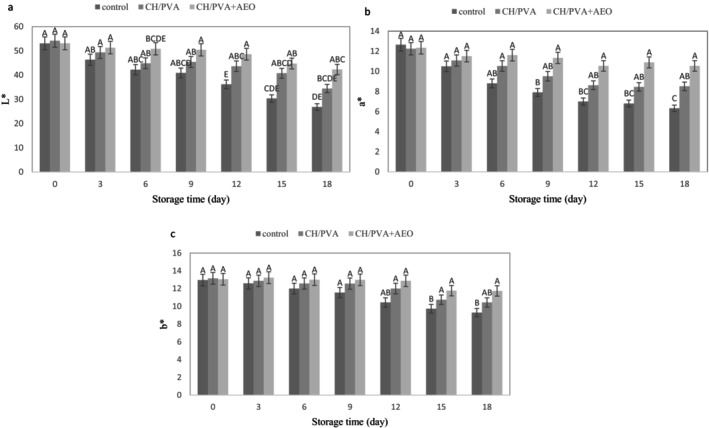
Changes in color parameters: L* (a), a* (b), b* (c) of minced beef packaged in packaging control, CH + PVA, CH + PVA + AEO during 18 days of storage at 4°C. Different letters indicate significant differences (*p* < 0.05).

Opacity and color are critical determinants in marketing strategies, influencing consumer perception and engagement through their psychological effects and visual appeal (Ghebleh and Sever 2020). The results of color changes for minced meat samples under different treatments stored at 4°C are presented in Figure 4. Brightness (L*) values decreased over the 18‐day storage period in all treatments, with the most significant decline (*p* < 0.05) observed in the control sample (Figure 4a). The reduction in brightness is associated with oxidation processes that alter the meat's color from red to dark brown (Konuk Takma and Korel 2019). The initial L* value for samples was recorded, on average, at 53.10. By the end of the 18th day, the L* values for the control, CH + PVA, and CH + PVA + AEO treatments were 26.85, 34.45, and 42.33, respectively, which indicated a significant difference between the control treatment and the CH + PVA + AEO treatment during storage time (*p* < 0.05). Farajzadeh et al. (2016) noted that the use of CH edible film along with gelatin reduces color change processes in packaging films containing shrimp ( *Litopenaeus vannamei* ).

The a* parameter, which indicates redness, is a crucial color parameter for meat and meat products (Ghebleh and Sever 2020). Throughout the 18 days of storage, a* values in all samples exhibited a decline (Figure 4b). According to El Adab and Hassouna (2016), this decrease in both a* and L* values signifies the oxidation of myoglobin and the subsequent formation of met‐myoglobin, resulting in a brown coloration of the meat (*p* < 0.05). The initial value of a* for samples was, on average, 12.41, while at the conclusion of the storage period, the control sample demonstrated the highest loss of redness (6.32); conversely, the treatment CH + PVA + AEO exhibited the lowest loss of a* (10.54). In related research, Konuk Takma and Korel (2019) found that incorporating black cumin essential oil into film made with chitosan and gelatin effectively preserves both L* and a* values in chicken breast meat, thereby delaying color loss. Additionally, Venkatachalam and Lekjing (2020) showed that chitosan coating with clove essential oil and nisin can delay the red (a*) changes of pork during storage through the antimicrobial and antioxidant activities of these compounds. Active packaging and nanofiber films infused with essential oils that possess antimicrobial and antioxidant properties can significantly (*p* < 0.05) slow down the formation of undesirable met‐myoglobin pigments, thus maintaining the quality of meat color over extended periods (Venkatachalam and Lekjing 2020).

The effect of treatments and storage time on the b* parameter (yellowness index) was not significant (*p* > 0.05); however, a slight decrease in b* value of all treatments was observed over the 18‐day refrigerated storage (Figure 4c). This decline in b* values is attributed to the reduction in oxymyoglobin content, resulting from oxygen consumption by microorganisms and the subsequent formation of metmyoglobin in meat (El Adab and Hassouna 2016). As illustrated in Figure 4c, the treatment CH + PVA+ AEO exhibited a smaller decrease in b* values by the end of the 18th day compared to the control sample. This phenomenon may be due to the antioxidant compounds and radical scavenging properties of AEO, which effectively delay oxidation (Karimkhani and Bagheri Sales 2021). In a related study, Alirezalu et al. (2022), reported that combining calcium‐alginate coating with AEO preserves the color of chicken breast meat samples. This coating acts as an oxygen barrier, effectively delaying myoglobin oxidation, particularly in formulations containing higher concentrations of AEO, which can mitigate oxidation by slightly reducing oxygen exposure.

#### 3.2.8 Sensory Evaluation

Evaluation of sensory characteristics such as texture, odor, color is one of the most important factors influencing product acceptance by consumers and can provide information about the freshness and quality of minced meat (Yaghoubi et al. 2021). The results of evaluating the sensory characteristics (appearance, texture, color, odor, and overall acceptability) of minced meat during 18 days of storage are presented in Table 1. The results indicated that, throughout the storage period, the control group consistently exhibited the lowest scores (*p* < 0.05). Panelists did not receive any negative sensory judgments when using a 2% concentration in the films made. In a similar study, Venkatachalam and Lekjing (2020) worked on chitosan film containing clove essential oil and nisin to improve the quality and shelf life of pork meat in cold storage. The results showed that with the increase in storage time, due to the growth of microorganisms and oxidation of lipids, the sensory characteristics of the product decreased. According to Table 1, at the beginning of the storage period, all the samples had an excellent score (8.9 to 9), and there was no significant difference (*p* > 0.05) between the samples. However, on the ninth day, the control samples obtained an unacceptable score (less than 5) in terms of color, texture, odor, appearance, and overall acceptance. In contrast, CH + PVA and CH + PVA + AEO treatments significantly (*p* < 0.05) delayed the loss of sensory scores. In a similar study, Esmaeili and Khodanazary (2021), reported that the use of pectin coating combined with CH and AEO delays the loss of sensory characteristics such as color, smell, and overall acceptance in fish meat. CH + PVA + AEO treatment was accepted until the 12th day, and the average sensory score of this treatment was 5.54. This could be attributed to the antimicrobial and antioxidant activity of CH and AEO, which effectively delayed the loss of sensory properties of minced meat (Venkatachalam and Lekjing 2020). Similar results were presented by Bagheri and Aryaee (2022), who stated that CH coating along with nettle essential oil and basil seed gum preserves the sensory characteristics (color and texture) of fresh hamburger. In another study, Sharafati Chaleshtori et al. (2016) found that the use of CH along with cumin and eucalyptus essential oils resulted in high overall acceptance and good taste in fresh chicken meat. Finally, according to the results of this study and for implementation in the food trade, it may be better to use films inserted on the inner surface of the packaging rather than being poured onto the surface of the minced meat.

**TABLE 1:** Changes sensory attributes (odor, color, texture, appearance, and overall acceptability) of minced beef packaged in packaging control, CH + PVA, CH + PVA + AEO during 18 days of storage at 4°C. Different letters indicate significant differences (*p* < 0.05).

Sensory attributes/day	Treatment	0	3	6	9	12	15	18
Appearance	Control	9 ± 0^A^	8.2 ± 0.57^ABC^	6.8 ± 0.45^E^	4.3 ± 0.43^IJ^	3.6 ± 0.27^JK^	0 ± 0^L^	0 ± 0^L^
	CH + PVA	9 ± 0^A^	8.7 ± 0.43^AB^	7.7 ± 0.43^CD^	6.7 ± 0.23^E^	5.4 ± 0.43^GH^	4 ± 0^IJK^	3.3 ± 0.14^K^
	CH + PVA + AEO	9 ± 0^A^	8.7 ± 0.12^AB^	8 ± 0^BC^	6.9 ± 0.11^DE^	6.2 ± 0.54^FG^	5.7 ± 0.37^FG^	4.7 ± 0.37^HI^
Color	Control	9 ± 0^A^	7.7 ± 0.32BC	6.3 ± 0.51^DE^	4 ± 0^G^	1.4 ± 0.27^H^	0 ± 0^I^	0 ± 0^I^
	CH + PVA	8.9 ± 0^A^	8.5 ± 0.61^A^	7.2 ± 0.81^C^	6.2 ± 0.43^DE^	5.3 ± 0.18^F^	4.1 ± 0.45^G^	3.6 ± 0.22^G^
	CH + PVA + AEO	8.9 ± 0^A^	8.8 ± 0.25^A^	8.2 ± 0.64^AB^	7.3 ± 0.16^C^	6.9 ± 0.25^CD^	5.5 ± 0.18^EF^	5.3 ± 0.54^F^
Odor	Control	8.9 ± 1.38^AB^	6.2 ± 0.43^DE^	3.6 ± 0.43^GH^	1.7 ± 0.52^I^	1.2 ± 0.23^I^	0 ± 0^J^	0 ± 0^J^
	CH + PVA	8.9 ± 0^A^	8.4 ± 0.70^A^	7 ± 0^CD^	5.6 ± 0.72^E^	4.4 ± 0.18^FG^	3.6 ± 0.33^GF^	3 ± 0^H^
	CH + PVA + AEO	8.9 ± 0^A^	8.7 ± 0.58^A^	7.5 ± 0.53BC	6.4 ± 0.43^DE^	5.7 ± 0.25^E^	4.5 ± 0.43^H^	3.8 ± 0.39^GH^
Texture	Control	9 ± 0^A^	8.7 ± 0.32^ABC^	5.7 ± 0.51^E^	4.3 ± 0.41^F^	3.5 ± 0.43^FG^	0 ± 0^H^	0 ± 0^H^
	CH + PVA	9 ± 0^A^	8.9 ± 0.17^AB^	8 ± 0^BC^	7.1 ± 0.32^D^	5.7 ± 0.62^E^	3.7 ± 0.51^FG^	3 ± 0^G^
	CH + PVA + AEO	9 ± 0^A^	8.9 ± 0^A^	8.7 ± 0.43^AB^	8.3 ± 0.23^ABC^	7.7 ± 0.51^CD^	5.3 ± 0.27^E^	3.9 ± 0.16^F^
Overall acceptability	Control	9 ± 0^A^	8 ± 0^B^	6.4 ± 0.51^DE^	4.6 ± 0.51^I^	3.2 ± 0.337^JK^	0 ± 0^L^	0 ± 0^L^
	CH + PVA	9 ± 0^A^	8.1 ± 0^B^	7.4 ± 0.19^C^	5.6 ± 0.46^FG^	5 ± 0^HI^	3.8 ± 0.16^J^	3.1 ± 0.21^K^
	CH + PVA + AEO	9 ± 0^A^	8.2 ± 0.54^B^	8 ± 0^B^	6.7 ± 0.47^D^	6.1 ± 0.32^EF^	5.4 ± 0.25^GH^	5 ± 0^HI^

## 4 Conclusion

In conclusion, this study demonstrates the significant potential of electrospun poly vinyl alcohol‐chitosan (CH + PVA) nanofibers encapsulated with *Artemisia essential oil* (AEO) as an innovative and sustainable film for active packaging in the preservation of minced beef. The incorporation of AEO into the nanofiber matrix not only enhanced the antioxidant and antimicrobial properties of the packaging but also provided controlled release of the essential oil, effectively protecting it from thermal and environmental degradation. The application of CH + PVA + AEO nanofibers in minced beef packaging significantly reduced microbial growth, lipid oxidation, and spoilage indicators such as PV, TVB‐N, and TBA, compared to the control. Additionally, the sensory evaluation revealed that the CH + PVA + AEO treatment maintained the color, texture, odor, and overall acceptability of the minced beef for an extended period, highlighting its effectiveness in preserving meat quality. This research underscores the importance of integrating natural essential oils, such as *Artemisia essential oil*, with biodegradable polymers like chitosan and PVA to develop eco‐friendly and efficient active packaging systems. The findings indicate that CH + PVA + AEO nanofibers offer a promising alternative to harmful and non‐biodegradable synthetic preservatives and respond to the growing demand for sustainable food preservation methods. Future studies could build upon these findings by analyzing specific microbial groups such as Enterobacteriaceae and yeasts/molds to provide an even more detailed mapping of the microbial ecology inhibited by the AEO‐loaded film. Furthermore, subsequent research could explore the scalability of this technology and its application to other perishable food products, utilizing more advanced analytical methods and tests for further advancing the field of active packaging and food safety.

## Author Contributions

**Amirhossein Nasiri:** writing – review and editing, writing – original draft, methodology, investigation, formal analysis, conceptualization. **Tayebeh Zeinali:** writing – review and editing, writing – original draft, conceptualization. **Elham Ansarifar:** writing – review and editing, writing – original draft, validation, supervision, project administration, methodology, investigation, data curation, conceptualization.

## Funding

This work was supported by Birjand University of Medical Sciences, 457373.

## Ethics Statement

This study was performed according to the international code of ethics.

## Conflicts of Interest

The authors declare no conflicts of interest.

## Data Availability

Data is presented in the paper. The grammatical and stylistic editing of the text was conducted using QuillBot and Perplexity AI for improving the readability and language of the manuscript.
